# Biosorbent Efficacy of Groundnut Husk for the Elimination of Chromium from the Effluent of Mojo Tannery Industry, Ethiopia

**DOI:** 10.1155/2022/9997348

**Published:** 2022-11-10

**Authors:** Kebru Tegegn, Zekeria Yusuf, J. M. Sasikumar, Kefelegn Gorfu

**Affiliations:** ^1^Department of Biology, Dire Dawa University, Dire Dawa, Ethiopia; ^2^School of Biological Sciences and Biotechnology, Haramaya University, Dire Dawa, Ethiopia; ^3^Central Laboratory, Haramaya University, Dire Dawa, Ethiopia

## Abstract

The present study aimed to assess the effects of raw (RGNH), ethanol-extracted (EEGNH), and acid-treated (ATGNH) groundnut (*Arachis hypogaea* L.) husk for removal of chromium from tannery effluent from the Mojo tannery industry, Ethiopia. The effects of biosorbent dose, agitation speed, and contact time of heavy metal on biosorbent were measured. The percentage removal of chromium was examined by a flame atomic absorption spectrophotometer (FAAS). Functional group characteristics of the biosorbents were analyzed using Fourier transform infrared spectra (FTIR). The results indicated that at low doses (2 g), acid-treated groundnut husk (ATGNH) exhibited the highest removal efficiency (89.93%), whereas at a high dose (4 g), the raw groundnut husk (RGNH) has a potential removal efficiency (91.03%). The least removal efficiency was displayed by ethanol-extracted groundnut husk (EEGNH) (65.43%) at a dose of 3 g. Relating to the contact time, the highest chromium removal (94.41%) was exhibited by ATGNH with a 1-hour contact time. However, as contact time increased from 2 to 4 hours, there was a general decrease in the efficiency of biosorption. The removal of chromium by RGNH and EEGNH increased as contact time increased from 1 to 3 hours. The highest chromium removal (90.02%) was observed with ATGNH at 160 rpm agitation speed.

## 1. Introduction

Heavy metals, with toxic and nongradable features, pose a foremost peril to the environment by causing contamination in river water through tannery, textile, food, fertilizer, and paper industries [1]. The effluents from these industries are the abode of heavy metals, xenobiotic compounds, petroleum products, dissolved particles, and other harmful materials. They expel heavy metals like chromium (Cr), cadmium (Cd), copper (Cu), lead (Pb), nickel (Ni), and zinc (Zn) by tanning processes. Heavy metals are metals with a high atomic weight and a reasonably high density that are extremely toxic [2]. These heavy metal pollutants have adverse effects on the environment and human health [3]. The usage of agro/food wastes and microbes as biosorbents for the removal of toxic heavy metals is being a promising technology [4–6].

Among the heavy metals, Cr is one of the most renowned pollutants, which is gaining attention due to its high toxicity even at low concentrations. Hence, it is imperative to devise techniques to eliminate Cr from the effluents before they are discharged into the water bodies. The conventional methods used for Cr removal were found to be inefficient and expensive when heavy metals are present in trace amounts [7]. Thus, it is vital to use eco-friendly, low cost, and effective biosorption methods to eradicate the chromium menace in aquatic environments. For Cr removal, biosorption practices using waste plant biomass and agro/food wastes have been reported [8]. Furthermore, biosorption may be useful for wastewater treatment in third-world countries like Ethiopia with low technology development [9].

In the wake of this, the present work involved using groundnut husk for the elimination of Cr from the tannery effluents. Groundnut has been one of the most important oilseed crops [10]. The groundnut is cultivated for food, cash, and animal feed in Ethiopia. It is primarily grown by smallholder farmers in the country's lowland and drought-prone areas [11]. Groundnut waste has been a tool to remove heavy metals from different effluents [12–14]. However, no work has been done on the optimization of the biosorbent potential of groundnut husks in Ethiopia. Therefore, the present study was undertaken to assess the biosorption potential of groundnut husks for the removal of chromium from the tannery effluent discharged into the Mojo River, Ethiopia.

## 2. Materials and Methods

### 2.1. Collection of Tannery Effluents

Effluent samples were collected from the Mojo tannery industry before they got mixed with river water. The Mojo tannery industry, located 75 kilometers south of Addis Ababa (the capital of Ethiopia), is a medium-sized Ethiopian leather industry with an installed capacity of processing 844,000 and 1,656,000 sheep and goat hides per year, respectively. The daily amount of wastewater discharged into the Mojo River ranges from 3500 to 5500 cubic meters [15].

### 2.2. Physicochemical Analysis of the Effluent

Before sampling, the plastic bottles were cleaned with water and rinsed with nitric acid. Samples were collected by sequential composite sampling using continuous, constant samples discharged at regular time intervals. The effluent samples were placed in an icebox to preserve them and transported to the Central Laboratory of Haramaya University for further analysis. The effluents were digested [16] with nitric acid (HNO_3_) and then subjected to measurement of total chromium content using flame atomic absorption spectrometry (Buck Scientific; 210 VGP) at 357.9 nm. The sorbent-free blank was used as the control. Potassium dichromate (K_2_Cr_2_O_7_) was used to prepare the calibration curve. In addition, electrical conductivity, pH, total dissolved solids (TDS), total lead, and total chromium in the tannery effluent were measured [17]. The color and odor of the effluent were evaluated by visual observation and comparison with a series of standard solutions.

### 2.3. Biosorbent Samples

Groundnut (*Arachis hypogaea* L.) husk samples were collected from Harar city, Ethiopia, and washed with distilled water to remove any contamination. After one day of drying, the husks were dried in an oven at 105°C for 24 h to a constant mass and ground to form a powder using a mechanical grinder at a size of 0.5 mm. The powder produced was referred to as raw groundnut husk (RGNH) and was then stored in airtight bottles and stored for further analysis. For acid treatment, 50 g of the RGNH was treated with 500 mL of 1 M of HCl for 2 hours with the shaking speed at 120 rpm. The acid-treated groundnut husk (ATGNH) was filtered and washed with distilled water until it reached a neutral pH and oven-dried at 105°C for 24 hours to remove excess moisture. The ethanol-extracted groundnut husk (EEGNH) was prepared by Soxhlet extracting RGNH (50 g) with ethanol (250 mL) for 16 hours. The resultant modified biosorbent, EEGNH, was kept in airtight bottles for further biosorption process and FTIR analysis [18].

### 2.4. Characterization of Raw and Modified Adsorbents

The characterization of adsorbents before and after biosorption was recorded by the FTIR spectrophotometer (Perkin Elmer Frontier; S. No. C109832) in the range of 4000 cm^−1^ to 400 cm^−1^ using a KBr disc for reference. For the formation of pellets, 0.002 g of groundnut husk was mixed with 0.3 g of KBr and pressed at 6–8 bar pressure [12].

### 2.5. Batch Biosorption Process

Batch biosorption experiments RGNH, ATGNH, and EEGNH were performed at room temperature (25°C) using various parameters such as the biosorbent dose (1–4 g), contact time with effluent (1–4 hours), and agitation speed (80–200 rpm) based on the procedure described by Liu [18] with slight modification. Before the experiment, a sample of the effluent was analyzed for the initial quantity of total chromium using flame atomic absorption spectroscopy (FAAS, Buck Scientific, 210 VGP, USA). Each plastic bottle was filled with 100 ml of digested effluent of a predetermined concentration of total chromium.

### 2.6. Effects of Dose, Contact Time, and Agitation Speed

The effects of biosorbent dose, contact time, and agitation speed were assessed based on the procedure followed by Deshmukh et al. [19] with some modifications. The effect of biosorbent dose on biosorption efficiency was studied by varying the dose of biosorbent from 1 to 4 g of RGNH, EEGNH, and ATGNH with an interval of 2 hours of contact time with 100 ml of digested effluent in 250 ml of plastic bottles and agitation speed at 160 rpm using a mechanical shaker. The pH was adjusted to 3.0 and the temperature was set to 25°C. After agitation for the scheduled contact time, the solutions were filtered, and the concentration of total Cr after biosorption was measured using FAAS. To study the effect of contact time on the biosorption efficiency of RGNH, EEGNH, and ATGNH, batch biosorption studies were performed in capped plastic bottles, and the samples were withdrawn at a time interval of 1 hour between 1 and 4 hours, while the dose and agitation speed were kept constant at 2 g for each biosorbent and 160 rpm, respectively. The effect of agitation speed (Mechanical Shaker–Julaba; SW22) on biosorption efficiency was investigated at 80, 120, 160, and 200 rpm for 2 hours of contact time, with 2 g of RGNH, EEGNH, and ATGNH in 100 ml of digested effluent at pH 3.0 and a temperature of 25°C [14].

The removal efficiency of biosorbents was calculated by the difference between the initial and final concentrations of total chromium before and after biosorption. The removal efficiency (RE) and biosorption capacity (qt) were evaluated using the following equations [19]:

(1) $$\begin{array}{c} Removal\;efficiency\left(RE\right)as\;a\;percentage=\frac{\left(Ci-Cf\right)x100}{Ci}\text{,} \end{array}$$

(2) $$\begin{array}{c} Biosorption\;capacity\left(qt\right)=\frac{\left(Co-Ce\right)xV}{M}\text{,} \end{array}$$

where *qt* is the uptake of total Cr in mg per g of biosorbent, V is the volume of metal solution in contact with the biosorbent in a liter, *C*_*o*_ and *Ce* are the initial and residual concentrations of metal in the solution in mg L^−1^, respectively, and *M* is the mass of biosorbent in gram.

### 2.7. Data Analysis

The experimental data in Table 1 data file are deposited as supplementary material (available here) and were analyzed using SAS version 9.2. FTIR charts were performed using Origin Pro 2018 version 95E for data analysis and graphing software. The mean comparison and analysis of variance (ANOVA) were carried out using the SAS version 9.2 software package. Statistically significant differences were indicated by *P* < 0.05.

## 3. Results and Discussion

### 3.1. Physicochemical Parameters of Tannery Effluent

The physicochemical parameters such as color, odor, pH, EC, TDS, total chromium, and total lead in the effluent were analyzed, and the results are reported in Table 1, along with the WHO [20] guidelines for water quality. According to Table 1, most of the measured parameters were found to be above the World Health Organization (WHO) standard. The concentration of total Cr was above the WHO guidelines. Any chemical concentration that exceeds 10% of WHO guidelines poses serious jeopardy to health [17].

### 3.2. Characterization of Raw and Modified Biosorbents

**Figure 1:**
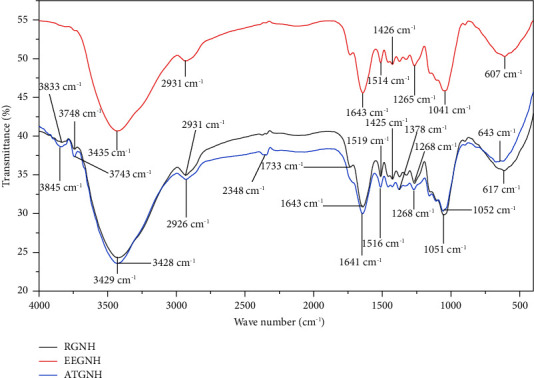
FTIR analysis of raw, ethanol-extracted, and acid-treated groundnut husk.

The FTIR spectra of RGNH, EEGNH, and ATGNH before Cr loading were measured at 400–4000 cm^−1^ and are shown in Figure 1. Several functional groups are revealed by FTIR spectrum analysis of RGNH, EEGNH, and ATGNH, indicating the complex nature of the biosorbent (Table 2). The functional groups viz. carboxyl, carbonyl, hydroxyl, phenyl, amino, and ester form complexes with metal ions by contributing an electron. The presence of hydroxyl groups, along with carbonyl groups, approved the presence of carboxylic acid groups in the biosorbent. A similar sort of spectra was attained for biosorption studies of metal ions using groundnut hull by [12]. Another study by Pangestu et al. [21] showed the role of carboxyl and amino groups in the metal adsorption process. This also designated the contribution of hydroxyl and carbonyl groups in the biosorption process of Cr as they are the most vital sorption sites [22].

**Figure 2:**
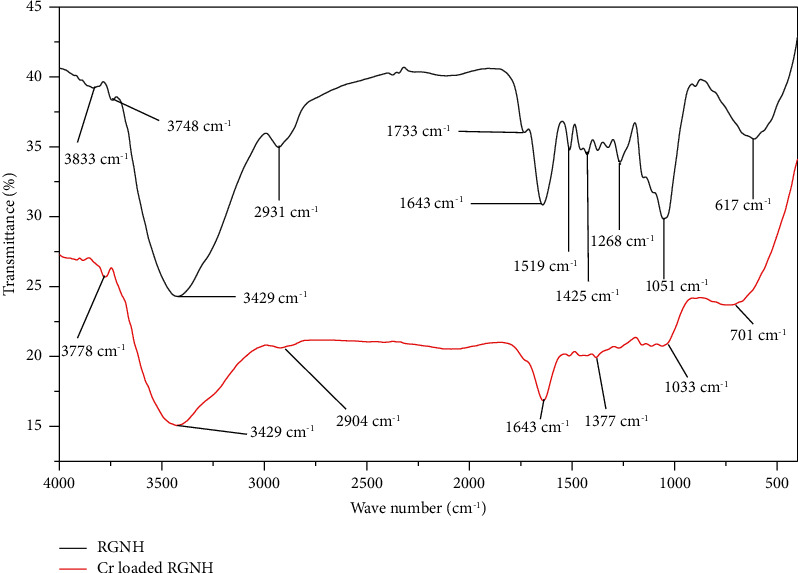
FTIR analysis of raw groundnut husk (RGNH) before and after Cr loaded.

**Figure 3:**
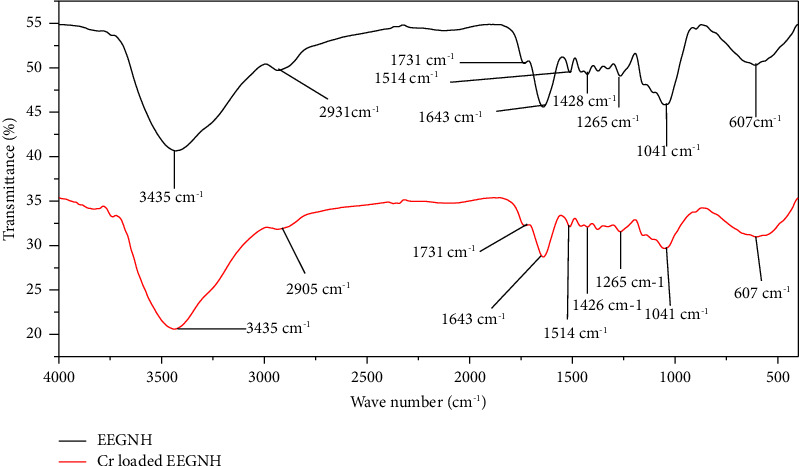
FTIR analysis of ethanol-extracted groundnut husk (EEGNH) before and after Cr loaded.

The FTIR spectrum analyses of RGNH, EEGNH, and ATGNH before and after sorption are shown (Table 2; Figures 2 and 3). FTIR spectrum analysis of biosorbent with chromium-loaded RGNH and ATGNH exhibited the broadening of the OH peak and -NH groups at 3475 cm^−1^, and OH groups bonded to methyl radical at 2910 cm^−1^ and peak 625 cm^−1^ and the peaks at 2348 cm^−1^ and 3748 cm^−1^ became broad (Figure 2), whereas in EEGNH, the peaks at 2348 cm^−1^ and 3748 cm^−1^ disappeared after biosorption (Figure 3). The broadening and loss of some peaks indicated the alteration of the structure of functional groups in the adsorbents is owing to the interaction of the surface functional groups [6, 23]. The observation of a new broader peak in ATGNH (Figure 3) at 3845 cm^−1^ after biosorption demonstrated its role in the biosorption of total Cr. This could be due to the treatment of the biosorbent with acid (HCl), which resulted in additional available binding sites at the surface of the biosorbent. This specified that ATGNH augmented its biosorption efficiency by creating an additional peak. This upsurge in the total Cr biosorption capacity of ATGNH might be owing to the removal of some minerals and organic matter and the exposure of available binding sites for metal biosorption. The study by [6] vindicated the role of acid pretreatment for the increased number of available binding sites on the surface of the biomass. The pattern of biosorption of metals onto biosorbent is attributable to the active groups and bonds present in them [24].

In this study, the removal efficiency of EEGNH was found to be lower than that of other sorbents. In EEGNH, disappearance of the peaks at 2348 cm^−1^ and 3748 cm^−1^ after biosorption was observed (Figure 3). This outcome showed that ethanol extraction of groundnut husk might have eliminated certain functional groups that are involved in biosorption, leading to a decreased biosorption efficiency of EEGNH. Therefore, modification of the biosorbent can increase or decrease the removal efficiency of biomass. According to [25], surface modifications (acidic and/or alkaline) enhanced the surface metal-binding capacity of the biosorbent.

### 3.3. Effect of Various Factors on Cr Sequestration

Table 3 presents the effect of biosorbent doses, agitation speeds, and contact times on the biosorbent potential of acid-treated (ATGNH), ethanol-extracted (EEGNH), and raw (RGNH) groundnut husk. The potential of a biosorbent is determined by the number of binding sites available to remove metal ions at a given initial metal ion concentration.

### 3.4. The Effect of the Biosorbent Dose

The findings of the study unveiled a dose-dependent efficiency of biosorption of Cr by RGNH, EEGNH, and ATGNH groundnut husks. The removal percentage increased significantly (*P* < 0.05) with the increase in dosages of the biosorbents. This may be due to the availability of more active sites on the surface or the greater availability of the surface area at a higher concentration of the adsorbent, resulting in a higher amount of metal uptake. A similar study was conducted by [26] which suggested that the groundnut shell biosorption efficiency for Cu and Pb was 68.2% and 77.8%, respectively. Groundnut shells are proved to be effective in removing toxic metals from zinc and chromium-plated water [27]. Romero et al. [21] indicated that the groundnut shell-activated carbon showed the highest adsorption of the chromium (IV) ions compared to the other metal ions.

Among the biosorbents used, AT groundnut husk exhibited significantly (*P* < 0.05) the highest removal efficiency (89.93%) at the dose of 2 g. This could be due to the availability of binding sites for biosorption of Cr. The results are supported by [25, 28], which also indicated that acid-treated biosorbent possessed the highest biosorption capacity. The RG (68.44%) and EE (65.43%) groundnut husks also displayed strong sorption of Cr at 3 g dosage. The removal rate of the biosorbents further declined as the dose increased, which indicated that the optimum biosorbent dosage was attained. This is also owing to a decrease in surface binding sites caused by partial biosorbent aggregation [29].

### 3.5. The Effect of Contact Time

The effect of contact time on the biosorption potential of biosorbents prepared from groundnut husks is shown in Table 3. The findings of the study revealed that the highest Cr sequestration (94.41%) and adsorption capacity of 0.58 mg/g were observed for ATGNH with a 1-hour contact time. On the other hand, the removal efficiency of RGNH and EEGNH increased as contact time increased from 1 to 3 hours. The removal effect of ATGNH decreased after 1 h of contact time. The fast initial uptake could be due to the rapid accumulation of the total chromium on the surface of the biosorbents. As binding sites become saturated with the contact time, biosorption will decrease. A similar trend was reported by [30, 31].

### 3.6. The Effect of Agitation Speed

The results in Table 3 revealed a consistent relationship between agitation speed and the sequester efficiency of the biosorbent. The highest Cr elimination (90.02%) was observed in ATGH at 160 rpm, whereas 89.43% of removal of Cr was exhibited by RGNH at 200 rpm. Agitation promotes the effective transfer of sorbent ions to sorbent sites by facilitating proper contact between metal ions in the solution and the biomass binding sites [32]. At moderate agitation, the contact between solid and liquid is enhanced. Previous research by [31] reported that the biosorption capacity of RGNH was high, measuring 0.42 mg/g and 0.55 mg/g at agitation speeds of 80 rpm and 200 rpm, respectively. The results also showed that the biosorption capacity of ATGNH was 0.42 mg/g and 0.55 mg/g at agitation speeds of 200 rpm and 160 rpm, respectively.

## 4. Conclusions

The findings of the present study unveiled a dose-dependent efficiency of biosorption of chromium (Cr) by raw groundnut, ethanol, and acid-treated groundnut husks. The removal percentage increased significantly with the increase in dosage of the biosorbents. This may be due to the availability of more active sites on the surface or greater availability of the surface area at a higher concentration of the adsorbent, resulting in a higher amount of metal uptake. The acid-treated and the untreated raw groundnut husks are proved to be potent biosorbents of chromium metal ions from wastewater. It was found that the biosorption efficiency was influenced by biosorbent dose, contact time, and agitation speed. That percentage of biosorption of Cr increased with an increase in biosorbent dose, contact time, and agitation speed.

## Figures and Tables

**Table 1:** Physicochemical characteristics of untreated tannery effluent.

Parameters	Detected value	WHO standard for drinking water (*a*)	NEQS limit discharge (*b*)	Remarks
Color	Blue-gray	Aesthetic value	—	—
Odor	Foul smell	Aesthetic only	—	—
pH	3.40	6.5–8.5	6–9	Exceed both *a* and *b*
TDS	1400 mg/L	600–1000 mg/L	3500	Exceed *a*
(EC)	1342 *μ*S/cm	Less than 400 *μ*S/cm	—	Exceed *a*
Total chromium (Cr)	12.25 mg/L	0.05 mg/L	1 mg/L	Exceed both *a* and *b*
Lead (Pb)	0.17 mg/L	0.01 mg/L	0.5 mg/L	Exceed *a*

**Table 2:** Functional groups of groundnut husk with or without chromium-loaded and the corresponding infrared absorption wavelengths.

Observed peak wavelength (cm^−1^)	Wavelength range (cm^−1^), intensity, and shape	Bond	Functional group assignment
3435–3364	3500–3200 (s, br)	O–H stretch, H–bonded	Alcohols, phenols
2931–2904	3000–2850 (m)	C–H stretch	Alkanes
1665–1641	1680–1620 (sat.),	C=C stretch	Alkenes
	1650–1600 (conj.) (w to m)		
1519–1514	1620–1440 (m to w)	C=C stretch	Aromatic compounds
1268–1265	1350–1000 (s to m)	C–N stretching	Amines
1052–1033	1050 (s)	S=O	Sulfoxide
701–607	700–610 (s, br)	–C≡C–H: C–H bend	Alkynes

**Table 3:** The effect of biosorbent dose, contact time, and agitation speed on chromium removal from tannery wastewater effluents.

Trt	Biosorbent dose (gm)	Initial concentration of Cr (mg/L)	Percentage removal of Cr	Agitation speed (rpm)	Percentage removal of Cr	Contact time (h)	Percentage removal of Cr
R	1.00	12.25a	28.68h	80.00	67.73b	1.00	39.06j
EE	1.00	12.25a	21.62i	80.00	20.11h	1.00	40.93i
AT	1.00	12.25a	67.73d	80.00	45.01ef	1.00	94.41a
R	2.00	12.25a	45.01g	120.00	60.08c	2.00	45.01h
EE	2.00	12.25a	43.94g	120.00	36.75g	2.00	43.94h
AT	2.00	12.25a	89.93a	120.00	47.63e	2.00	89.85b
R	3.00	12.25a	68.44d	160.00	46.21ef	3.00	56.39e
EE	3.00	12.25a	65.43e	160.00	44.34f	3.00	53.26f
AT	3.00	12.25a	85.84b	160.00	43.78f	3.00	80.61d
R	4.00	12.25a	91.03a	200.00	89.43a	4.00	53.18f
EE	4.00	12.25a	60.24f	200.00	53.44d	4.00	48.69g
AT	4.00	12.25a	83.27c	200.00	52.76d	4.00	84.78c

Means followed by same letter within a column were not significantly different at 0.05 probability level based on the LSD (least significance difference) test. *R*, powdered raw/untreated groundnut husk; EE, ethanol-extracted biosorbent; AT, acid-treated biosorbent.

## Data Availability

The data used to support the findings of this study are included within supplementary information file.

## Supplementary Materials

Supplementary Materials The biosorption data file in supplementary Table 1 is deposited as supplementary material.
