# Supplementary material for: Biosorbent Efficacy of Groundnut Husk for the Elimination of Chromium from the Effluent of Mojo Tannery Industry, Ethiopia
Source: Int J Biomater. 2022 Nov 10;2022:9997348. doi: 10.1155/2022/9997348 (PMC9671745; doi:10.1155/2022/9997348)
Supplement: Supplementary Materials — The biosorption data file in supplementary Table 1 is deposited as supplementary material. [file 9997348.f1.pdf]

Supplemnetary Table 1. Effects of biosorbent dose, contact time, and agitation speed

| trt | rep | dose(gm) | Agitation speed (rpm) | contact time(Hr.) |
|---|---|---|---|---|
| A1 | 1 | 1 | 160 | 2 |
| A1 | 2 | 1 | 160 | 2 |
| B1 | 1 | 1 | 160 | 2 |
| B1 | 2 | 1 | 160 | 2 |
| C1 | 1 | 1 | 160 | 2 |
| C1 | 2 | 1 | 160 | 2 |
| A2 | 1 | 2 | 160 | 2 |
| A2 | 2 | 2 | 160 | 2 |
| B2 | 1 | 2 | 160 | 2 |
| B2 | 2 | 2 | 160 | 2 |
| C2 | 1 | 2 | 160 | 2 |
| C2 | 2 | 2 | 160 | 2 |
| A3 | 1 | 3 | 160 | 2 |
| A3 | 2 | 3 | 160 | 2 |
| B3 | 1 | 3 | 160 | 2 |
| B3 | 2 | 3 | 160 | 2 |
| C3 | 1 | 3 | 160 | 2 |
| C3 | 2 | 3 | 160 | 2 |
| A4 | 1 | 4 | 160 | 2 |
| A4 | 2 | 4 | 160 | 2 |
| B4 | 1 | 4 | 160 | 2 |
| B4 | 2 | 4 | 160 | 2 |
| C4 | 1 | 4 | 160 | 2 |
| C4 | 2 | 4 | 160 | 2 |
| A21 | 1 | 2 | 160 | 1 |
| A21 | 2 | 2 | 160 | 1 |
| B21 | 1 | 2 | 160 | 1 |
| B21 | 2 | 2 | 160 | 1 |
| C21 | 1 | 2 | 160 | 1 |
| C21 | 2 | 2 | 160 | 1 |
| A22 | 1 | 2 | 160 | 2 |
| A22 | 2 | 2 | 160 | 2 |
| B22 | 1 | 2 | 160 | 2 |
| B22 | 2 | 2 | 160 | 2 |
| C22 | 1 | 2 | 160 | 2 |
| C22 | 2 | 2 | 160 | 2 |
| A23 | 1 | 2 | 160 | 3 |
| A23 | 2 | 2 | 160 | 3 |
| B23 | 1 | 2 | 160 | 3 |
| B23 | 2 | 2 | 160 | 3 |
| C23 | 1 | 2 | 160 | 3 |
| C23 | 2 | 2 | 160 | 3 |
| A24 | 1 | 2 | 160 | 4 |
| A24 | 2 | 2 | 160 | 4 |
| B24 | 1 | 2 | 160 | 4 |
| B24 | 2 | 2 | 160 | 4 |
| C24 | 1 | 2 | 160 | 4 |
| C24 | 2 | 2 | 160 | 4 |

| | | | | |
|---|---|---|---|---|
| A160 | 1 | 2 | 160 | 2 |
| A160 | 2 | 2 | 160 | 2 |
| B160 | 1 | 2 | 160 | 2 |
| B160 | 2 | 2 | 160 | 2 |
| C160 | 1 | 2 | 160 | 2 |
| C160 | 2 | 2 | 160 | 2 |
| A200 | 1 | 2 | 200 | 2 |
| A200 | 2 | 2 | 200 | 2 |
| B200 | 1 | 2 | 200 | 2 |
| B200 | 2 | 2 | 200 | 2 |
| C200 | 1 | 2 | 200 | 2 |
| C200 | 2 | 2 | 200 | 2 |
| A80 | 1 | 2 | 80 | 2 |
| A80 | 2 | 2 | 80 | 2 |
| B80 | 1 | 2 | 80 | 2 |
| B80 | 2 | 2 | 80 | 2 |
| C80 | 1 | 2 | 80 | 2 |
| C80 | 2 | 2 | 80 | 2 |
| A120 | 1 | 2 | 120 | 2 |
| A120 | 2 | 2 | 120 | 2 |
| B120 | 1 | 2 | 120 | 2 |
| B120 | 2 | 2 | 120 | 2 |
| C120 | 1 | 2 | 120 | 2 |
| C120 | 2 | 2 | 120 | 2 |

| initial con of Total Cr(mg/L) | after bio sorption | percentage removal |
|---|---|---|
| 12 | 8.67 | 27.75 |
| 12.5 | 8.8 | 29.6 |
| 12 | 9.5 | 20.83333333 |
| 12.5 | 9.7 | 22.4 |
| 12 | 4 | 66.66666667 |
| 12.5 | 3.9 | 68.8 |
| 12 | 6.67 | 44.41666667 |
| 12.5 | 6.8 | 45.6 |
| 12 | 6.83 | 43.08333333 |
| 12.5 | 6.9 | 44.8 |
| 12 | 1.17 | 90.25 |
| 12.5 | 1.3 | 89.6 |
| 12 | 3.83 | 68.08333333 |
| 12.5 | 3.9 | 68.8 |
| 12 | 4.17 | 65.25 |
| 12.5 | 4.3 | 65.6 |
| 12 | 1.67 | 86.08333333 |
| 12.5 | 1.8 | 85.6 |
| 12 | 1 | 91.66666667 |
| 12.5 | 1.2 | 90.4 |
| 12 | 4.83 | 59.75 |
| 12.5 | 4.91 | 60.72 |
| 12 | 2 | 83.33333333 |
| 12.5 | 2.1 | 83.2 |
| 12 | 7.33 | 38.91666667 |
| 12.5 | 7.6 | 39.2 |
| 12 | 7.17 | 40.25 |
| 12.5 | 7.3 | 41.6 |
| 12 | 0.67 | 94.41666667 |
| 12.5 | 0.7 | 94.4 |
| 12 | 6.67 | 44.41666667 |
| 12.5 | 6.8 | 45.6 |
| 12 | 6.83 | 43.08333333 |
| 12.5 | 6.9 | 44.8 |
| 12 | 1.17 | 90.25 |
| 12.5 | 1.32 | 89.44 |
| 12 | 5.33 | 55.58333333 |
| 12.5 | 5.35 | 57.2 |
| 12 | 5.67 | 52.75 |
| 12.5 | 5.78 | 53.76 |
| 12 | 2.33 | 80.58333333 |
| 12.5 | 2.42 | 80.64 |
| 12 | 5.67 | 52.75 |
| 12.5 | 5.8 | 53.6 |
| 12 | 6.17 | 48.58333333 |
| 12.5 | 6.4 | 48.8 |
| 12 | 1.83 | 84.75 |
| 12.5 | 1.9 | 84.8 |

| | | |
|---|---|---|
| 12.5 | 6.5 | 48 |
| 12 | 6.67 | 44.41666667 |
| 12.5 | 6.8 | 45.6 |
| 12 | 6.83 | 43.08333333 |
| 12.5 | 6.9 | 44.8 |
| 12 | 6.87 | 42.75 |
| 12.5 | 1.3 | 89.6 |
| 12 | 1.29 | 89.25 |
| 12.5 | 5.91 | 52.72 |
| 12 | 5.5 | 54.16666667 |
| 12.5 | 5.8 | 53.6 |
| 12 | 5.77 | 51.91666667 |
| 12.5 | 3.9 | 68.8 |
| 12 | 4 | 66.66666667 |
| 12 | 9.67 | 19.41666667 |
| 12.5 | 9.9 | 20.8 |
| 12 | 6.67 | 44.41666667 |
| 12.5 | 6.8 | 45.6 |
| 12 | 4.83 | 59.75 |
| 12.5 | 4.95 | 60.4 |
| 12 | 7.5 | 37.5 |
| 12.5 | 8 | 36 |
| 12 | 6.33 | 47.25 |
| 12.5 | 6.5 | 48 |
